# Genetic Variants Associated With Cancer Therapy–Induced Cardiomyopathy

**DOI:** 10.1161/CIRCULATIONAHA.118.037934

**Published:** 2019-04-16

**Authors:** Pablo Garcia-Pavia, Yuri Kim, Maria Alejandra Restrepo-Cordoba, Ida G. Lunde, Hiroko Wakimoto, Amanda M. Smith, Christopher N. Toepfer, Kelly Getz, Joshua Gorham, Parth Patel, Kaoru Ito, Jonathan A. Willcox, Zoltan Arany, Jian Li, Anjali T. Owens, Risha Govind, Beatriz Nuñez, Erica Mazaika, Antoni Bayes-Genis, Roddy Walsh, Brian Finkelman, Josep Lupon, Nicola Whiffin, Isabel Serrano, William Midwinter, Alicja Wilk, Alfredo Bardaji, Nathan Ingold, Rachel Buchan, Upasana Tayal, Domingo A. Pascual-Figal, Antonio de Marvao, Mian Ahmad, Jose Manuel Garcia-Pinilla, Antonis Pantazis, Fernando Dominguez, A. John Baksi, Declan P. O’Regan, Stuart D. Rosen, Sanjay K. Prasad, Enrique Lara-Pezzi, Mariano Provencio, Alexander R. Lyon, Luis Alonso-Pulpon, Stuart A. Cook, Steven R. DePalma, Paul J.R. Barton, Richard Aplenc, Jonathan G. Seidman, Bonnie Ky, James S. Ware, Christine E. Seidman

**Affiliations:** 1 Hospital Universitario Puerta de Hierro, Madrid, Spain (P.G.-P., M.A.R.-C., F.D., L.A.-P.).; 2 Centro de Investigación Biomédica en Red Enfermedades in Cardiovascular Diseases (CIBERCV), Madrid, Spain (P.G.-P., M.A.R.-C., A.B.-G., J. Lupon, D.A.P.-F., J.M.G.-P., F.D., E.L.-P., L.A.-P.).; 3 University Francisco de Vitoria, Pozuelo de Alarcón, Madrid, Spain (P.G.-P.).; 4 Harvard Medical School, Boston, MA (Y.K., I.G.L., H.W., C.N.T., J.G., P.P., K.I., J.A.W., S.R.D., J.G.S., C.E.S.).; 5 Massachusetts General Hospital, Boston (Y.K.).; 6 Oslo University Hospital and University of Oslo, Norway (I.G.L.).; 7 Perelman School of Medicine and University of Pennsylvania Health System, Philadelphia (A.M.S., K.G., Z.A., J. Li, A.T.O., B.F., R.A., B.K.).; 8 University of Oxford (C.N.T.).; 9 National Heart & Lung Institute, Imperial College London, UK (R.G., E.M., R.W., N.W., W.M., A.W., N.I., R.B., U.T., A.d.M., M.A., A.P., A.J.B., S.D.R., S.K.P., E.L.-P., A.R.L., S.A.C., P.J.R.B., J.S.W.).; 10 Royal Brompton & Harefield NHS Foundation Trust, London, UK (R.G., E.M., R.W., N.W., W.M., A.W., N.I., R.B., U.T., A.d.M., M.A., A.P., A.J.B., S.D.R., S.K.P., A.R.L., P.J.R.B., J.S.W.).; 11 Hospital Universitario Puerta de Hierro, Universidad Autónoma de Madrid, Spain (B.N., M.P.).; 12 Hospital Universitario Germans Trias i Pujol, Badalona, Spain (A.B.-G., J. Lupon).; 13 MRC London Institute of Medical Sciences, Imperial College UK (N.W., D.P.O., S.A.C., J.S.W., C.E.S., A.d.M.).; 14 Hospital Universitario de Tarragona Joan XXIII. IISPV, Rovira Virgili University, Spain (I.S., A.B.).; 15 Hospital Universitario Virgen de la Arrixaca, University of Murcia. Spain (D.A.P.-F.).; 16 Centro Nacional de Investigaciones Cardiovasculares, Madrid, Spain (E.L.-P.).; 17 National Heart Centre Singapore and Duke-National University of Singapore (S.A.C.).; 18 Howard Hughes Medical Institute, Chevy Chase, MD (S.R.D., C.E.S.).; 19 Brigham and Women's Hospital, Boston MA (P.P., C.E.S.).; 20 Hospital Universitario Virgen de la Victoria, IBIMA, Malaga, Spain (J.M.G.-P.).

**Keywords:** cardiomyopathies, drug therapy, genetics, medical oncology, titin

## Abstract

**Background:**

Cancer therapy–induced cardiomyopathy (CCM) is associated with cumulative drug exposures and preexisting cardiovascular disorders. These parameters incompletely account for substantial interindividual susceptibility to CCM. We hypothesized that rare variants in cardiomyopathy genes contribute to CCM.

**Methods:**

We studied 213 patients with CCM from 3 cohorts: retrospectively recruited adults with diverse cancers (n=99), prospectively phenotyped adults with breast cancer (n=73), and prospectively phenotyped children with acute myeloid leukemia (n=41). Cardiomyopathy genes, including 9 prespecified genes, were sequenced. The prevalence of rare variants was compared between CCM cohorts and The Cancer Genome Atlas participants (n=2053), healthy volunteers (n=445), and an ancestry-matched reference population. Clinical characteristics and outcomes were assessed and stratified by genotypes. A prevalent CCM genotype was modeled in anthracycline-treated mice.

**Results:**

CCM was diagnosed 0.4 to 9 years after chemotherapy; 90% of these patients received anthracyclines. Adult patients with CCM had cardiovascular risk factors similar to the US population. Among 9 prioritized genes, patients with CCM had more rare protein-altering variants than comparative cohorts (*P*≤1.98e–04). Titin-truncating variants (TTNtvs) predominated, occurring in 7.5% of patients with CCM versus 1.1% of The Cancer Genome Atlas participants (*P*=7.36e–08), 0.7% of healthy volunteers (*P*=3.42e–06), and 0.6% of the reference population (*P*=5.87e–14). Adult patients who had CCM with TTNtvs experienced more heart failure and atrial fibrillation (*P*=0.003) and impaired myocardial recovery (*P*=0.03) than those without. Consistent with human data, anthracycline-treated TTNtv mice and isolated TTNtv cardiomyocytes showed sustained contractile dysfunction unlike wild-type (*P*=0.0004 and *P*<0.002, respectively).

**Conclusions:**

Unrecognized rare variants in cardiomyopathy-associated genes, particularly TTNtvs, increased the risk for CCM in children and adults, and adverse cardiac events in adults. Genotype, along with cumulative chemotherapy dosage and traditional cardiovascular risk factors, improves the identification of patients who have cancer at highest risk for CCM.

**Clinical Trial Registration:**

URL: https://www.clinicaltrials.gov. Unique identifiers: NCT01173341; AAML1031; NCT01371981.

## Abstract 2

Supplemental Digital Content is available in the text.

**Clinical Perspective**

**What Is New?**

- This is the first study to consider the association between rare genetic variants in a large set of cardiomyopathy genes and the occurrence of cancer therapy–induced cardiomyopathy (CCM).
- We demonstrated an increased prevalence of rare variants in cardiomyopathy genes, in particular, truncating variants in the TTN gene, in adult and pediatric patients who have cancer with CCM.
- We confirmed human genetic data with experimental analyses, showing that anthracyclines induced protracted left ventricular dysfunction in mice with titin-truncating variants, but not in wild-type mice.

**What Are the Clinical Implications?**

- Our findings show that variants in cardiomyopathy genes contribute to CCM susceptibility among adult and pediatric patients with cancer.
- The identification of genetic risk factors opens new opportunities to define patients at high risk for CCM and associated adverse outcomes.
- Future investigations to define patients who have cancer with high risk for CCM through genetic testing can assess the efficacy of prophylactic cardioprotective drugs and treatment regimens to reduce CCM while providing effective cancer therapy.

Considerable advances in cancer therapies have led to major improvements in long-term survival for many malignancies, but also to unintended side effects, including cardiotoxicity.^1,2^ Cancer therapy–induced cardiomyopathy (CCM), identified as reduced left ventricular ejection fraction (LVEF) with or without signs and symptoms of overt heart failure,^3^ can occur during, shortly after, or many years beyond cancer treatments and affects the long-term prognosis of patients.^1,4,5^

Anthracyclines, which are commonly used to treat both solid tumors and hematologic malignancies in children and adults,^2^ cause cardiotoxicity in up to 10% of patients with cumulative dosages of 250 mg/m^2^ but in 65% of patients receiving cumulative dosages >550 mg/m^2^.^6^ Combining anthracyclines with other therapies, such as trastuzumab (an antibody targeting HER-2), can provoke greater cardiotoxicity with depressed LVEF occurring in ≈18% to 34% of treated individuals, and severe, symptomatic heart failure in 2% to 4%.^3,7^ Additional clinical parameters are recognized to contribute to CCM, including female sex, extremes of age, and preexisting cardiac risk factors.^2^ Even when accounting for these factors, predicting individual susceptibility to CCM remains challenging.

Several candidate gene and genome-wide association studies have identified common genetic variants that are associated with CCM through candidate gene analyses and genome-wide association studies.^2,8–12^ Although a recent systemic literature review concluded that the overall evidence supporting variant associations with CCM was limited, genetic data were robust for one intergenic variant (rs28714259) and variants in proximity to 4 other genes.^13^ Rare variants in genes that cause familial cardiomyopathies^14^ have also been identified in several small case series and isolated patients with CCM.^13,15–17^

To better understand the clinical and genetic determinants in CCM, we studied 3 CCM cohorts comprising adult and pediatric patients with diverse malignancies, of whom 90% received anthracyclines. We then corroborated our human findings through cardiac phenotyping of anthracycline-treated mice. From these analyses, we demonstrate the direct and prevalent involvement of variants in genes associated with dilated cardiomyopathy and, in particular, titin-truncating variants (TTNtvs) in CCM.

## Methods

The data that support the findings of this study are available within the article, the online supplementary files, and publicly available databases. Additional requests, from qualified researchers trained in human subject confidentiality protocols, for anonymized data may be sent to the corresponding authors.

### CCM Cohorts, Healthy Volunteer, and Population Controls

Research protocols were reviewed and approved by the institutional ethics board at each participating site. Adult patients with CCM (cohorts A and B), parents of minor patients with CCM (cohort C), and healthy volunteers provided written informed consent. Cohort A includes non-Finnish European patients with CCM retrospectively collected from 6 European heart failure or cardiac transplantation clinics in Spain and the United Kingdom. Cohort B includes prospectively enrolled patients with breast cancer, participating in cardiotoxicity studies of cancer treatments (clinicaltrials.gov NCT01173341). Cohort C includes pediatric patients with newly diagnosed acute myeloid leukemia, enrolled in a clinical therapy trial therapy (AAML1031; clinicaltrials.gov NCT01371981). Cohorts B and C are US patients with non-Finnish European, African, or Asian ancestry, who had prespecified clinical assessments with cardiac imaging (echocardiograms or multigated acquisition scans) before, during, and after chemotherapy. Table 1 provides additional demographic profiles on these cohorts.

**Table 1.**
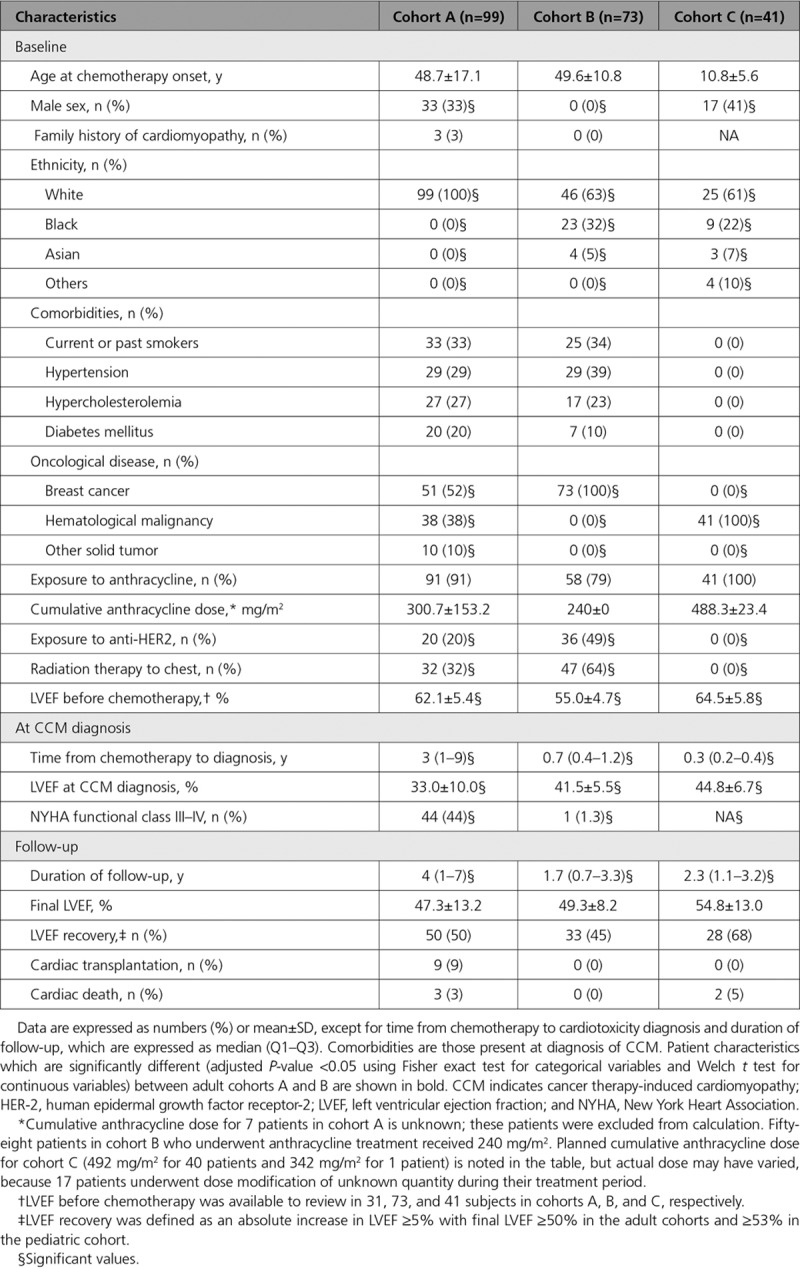
Clinical Characteristics at Baseline and Follow-Up in Patients With CCM in Study Cohorts

CCM was diagnosed irrespective of symptoms based on LVEF to <50 (cohort B) or <53% (cohorts A and C)^3,18,19^ and ≥10% reduction from baseline by echocardiography or <50% and ≥10% reduction from baseline by radionuclide ventriculography, in the absence of established coronary artery disease, cardiomyopathy, primary valvular disease, or uncontrolled hypertension. Additional clinical information including follow-up duration and adverse outcomes was obtained from medical and clinical trial records and patient reports. Where prechemotherapy cardiac imaging was absent, patients were included when LVEF was ≤45% and no alternative cause for cardiac dysfunction other than chemotherapy was identified. LVEF recovery was defined by a final LVEF ≥50% with ≥5% LVEF increase or restoration of LVEF to the baseline value.^18^

Healthy volunteers of European ancestry (n=445) were prospectively recruited participants into the U.K. Digital Heart Project (https://digital-heart.org/)^20^ with no cardiovascular disease or risk factors by self-report and normal cardiac magnetic resonance imaging.

### Next-Generation Sequencing and Variant Analysis

Genomic DNA extracted from peripheral blood samples was used to produce DNA-sequencing libraries that were captured and sequenced on Illumina TruSight Cardio Sequencing kit and a custom Agilent array (DCMv5) as described.^20,21^ Variants were identified using the Genome Analysis Took Kit (GATK) HaplotypeCaller tool following GATK Best Practices.^22^ Rare variants (minor allele frequency <1.0e–4, assessed in ancestry-matched subjects in the Genome Aggregation Database [gnomAD]^23^) were annotated by SnpEFF and GRCh37.68 (also see the online-only Data Supplement). The cumulative burden of rare variants in cardiomyopathy genes was compared in CCM cohorts with all patients who had breast (n=1042) and lung (n=1011) cancer participating in The Cancer Genome Project (TCGA),^24^ healthy volunteers, and gnomAD^23^ subjects with non-Finnish European, African, and Asian ancestries (combined and in ancestry-specific analyses).

### Anthracycline Treatment of Mice

Protocols were reviewed and approved by the Institutional Animal Care and Use Committee at Harvard Medical School (Boston, MA). Wild-type and heterozygous C57BL/6N mice with a titin A-band truncation (Ttn^tv/+^)^25,26^ received 3 doses of intraperitoneal doxorubicin (5 mg/kg) at weekly intervals (≈45 mg/m^2^). Cardiac function was assessed in vivo at baseline (age=10–14 weeks) and weekly using a digital ultrasound system (Vevo 2100 Imaging System and MS550D transducer; FujiFilm VisualSonics) by an experienced observer blinded to mouse genotype and treatment. Cardiomyocytes from treated and untreated wild-type and Ttn^tv/+^ mice were isolated and sarcomere contractility was measured (see the online-only Data Supplement Methods).

### Statistical Analyses

Cohort and subgroup analyses, and comparisons with TCGA genomic data,^24^ healthy volunteers, and reference populations^23^ were performed by Fisher exact test (2-tailed), binomial test, or Pearson χ^2^ test of association for categorical values. Welch *t* test and Kruskal-Wallis rank sum tests were used to assess numerical data. Analyses were conducted using either the Stata SE package (version 14, StataCorp) or the R statistical package (version 3.4.0; http://www.R-project.org/).

Additional method details are provided in the online-only Data Supplement.

## Results

### Patients With CCM

We studied 3 CCM cohorts (Table 1). Cohort A includes 99 patients of European ancestry with hematologic, breast, or other solid-tumor cancer (mean age at treatment=48.7±17.1 years), recruited from heart failure and cardiac transplant clinics. Two US cohorts were identified through prospective longitudinal cardiac evaluations obtained throughout cancer therapy: Cohort B comprised 73 patients (mean age at treatment=49.6±10.8 years) with European, African, or Asian ancestry, enrolled from breast cancer clinics as part of a prospective study of who developed CCM during treatment; Cohort C comprised 41 pediatric patients with newly diagnosed acute myelogenous leukemia (mean age at treatment=10.8±5.6 years) of diverse ancestries. Although individual treatments varied, 90% of all patients with CCM received anthracycline and 33% of adults received trastuzumab. After normalizing anthracycline doses^27^ the cumulative equivalent dose was <400 mg/m^2^ in 93.9% of patients in cohort A, 100% of patients in cohort B, and 2.3% of patients in cohort C.

We assessed clinical risk factors for CCM in these cohorts. Seventy-six percent of all patients were CCM were females, predominantly treated for breast cancer. In cohorts A and B the prevalence of cigarette smoking, hypertension, and diabetes mellitus was comparable (*P*=not significant) to that of the general US population,^28^ but hypercholesterolemia in patients with cancer was less common (*P*=3.0e–09). Three patients in cohort A, without prechemotherapy imaging studies, had family histories of cardiomyopathy of unknown cause. Patients in cohort C were considerably younger (mean age=10.8±5.6 years), without cardiovascular risk factors, and all had normal LVEF at study entry.

The median time after the initiation of cancer treatment to CCM diagnosis in cohort A was 3.0 (range=1–9) years, but 0.3 to 0.7 years for cohorts B and C because of cardiac surveillance during treatment in these 2 cohorts. At CCM diagnosis, the mean LVEF decrease was 23.4±9.2% in cohort A, 13.5±3.3% in cohort B, and 19.7±6.0% in cohort C. Across all cohorts, treatment with high cumulative anthracycline dose (>400 mg/m^2^) was not associated with poorer left ventricular (LV) dysfunction at CCM diagnosis (mean LVEF=42.0±9.6%). Patients (cohorts A and B) who received trastuzumab without anthracycline had similar cardiovascular risk factors and no significant differences in either baseline or postchemotherapeutic LVEF (mean LVEF decrease=13.9±3.6%) in comparison with patients receiving anthracyclines with or without other agents (mean LVEF decrease=16.7±7.5%). Cardiac recovery occurred in approximately half of patients with CCM from each cohort, but 9% of patients in cohort A underwent cardiac transplantation. Cardiac deaths occurred in 3% of patients in cohort A and in 5% of patients in cohort C.

### Gene Variants in Patients With CCM

We previously identified 9 genes with an excess of rare missense and in-frame insertions/deletion or truncating variants among patients with cardiomyopathy.^29^ Within these prespecified genes, we examined rare variants (defined as minor allele frequency <1.0e–4) among ancestry-matched reference populations,^23^ CCM cohorts, healthy volunteers, and all breast and lung cancer participants in TCGA^24^ (Table 2). Because anthracyclines are highly effective and widely used to treat these malignancies,^6^ we expect that most TCGA participants received this chemotherapeutic agent. The prevalence of rare protein-altering variants across all 9 genes was significantly higher in a combined CCM cohort than in unselected lung and breast cancer TCGA participants (*P*=1.98e–04), healthy volunteers (*P*=3.90e–05), and reference populations (*P*=1.78e–06). Although patients with CCM had rare variants in several established dilated cardiomyopathy (DCM) genes (*BAG3*, *LMNA*, *MYH7*, *TCAP*, *TNNT2*, and *TTN*), only variants in *TTN*, which encodes titin, were significantly increased. TTNtvs were highly enriched in all patients with CCM (n=16; 7.5%) in comparison with unselected breast or lung TCGA participants (combined, *P*=7.36e–08 and Table I in the online-only Data Supplement), healthy volunteers (*P*=3.42e–06), and the reference population (*P*=5.87e–14). Subanalyses of patients with CCM and the reference population stratified by ancestry (Table II in the online-only Data Supplement), although limited by small numbers, confirmed the observed enrichment of TTNtvs in all patients with CCM. TTNtvs that are significantly increased in patients with DCM ^20,30^ reside in exons that are highly expressed in LV tissues, especially those that encode the A-band and distal I-band.^31^ TTNtvs identified in patients with CCM shared these characteristics (Table 3).

**Table 2.**
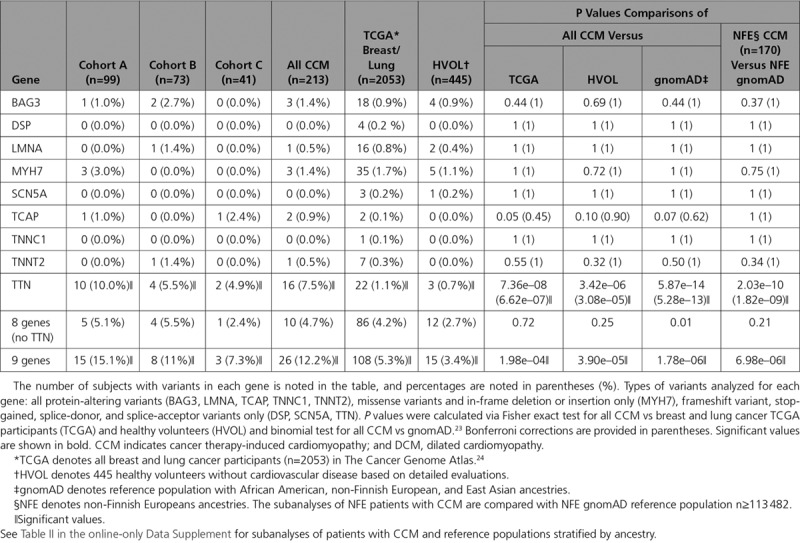
Burden Analysis of 9 DCM Genes in CCM Cohorts

**Table 3.**
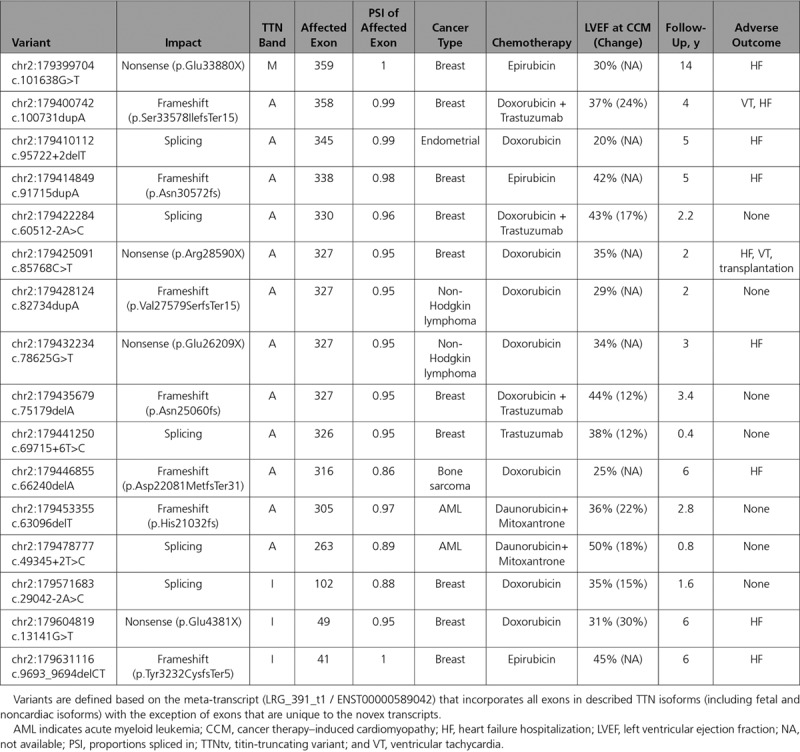
Summary of TTNtv Identified From All 3 Cohorts With CCM

We extended these analyses to include 40 other genes that have been implicated in cardiomyopathies.^29^ Variants in these genes account for a very small fraction of unselected patients with cardiomyopathy. There was no significant difference in the prevalence of all rare protein-altering variants (minor allele frequency <1.0e–4; Tables III and IV in the online-only Data Supplement) or variants predicted as damaging (Tables V and VI in the online-only Data Supplement) in patients with CCM in each cohort or the combined CCM cohort, in comparison with healthy volunteers or in the reference population. For individual genes, the prevalence of rare variants was nominally increased only in *FKRP* (encoding fukutin-related protein); recessive *FKRP* mutations cause several forms of muscular dystrophies with cardiac involvement.^32^

### Clinical Outcomes in Adult Patients Who Have CCM With or Without TTNtvs

Patients with CCM in cohorts A and B were predominantly women (81%), with breast cancer (73%), with traditional cardiovascular risk factors, who received anthracyclines (86.6%) or trastuzumab (33%), and with follow-up between 8.4 months and 18 years (Table 1). We defined the clinical courses among patients who have CCM with TTNtv and compared risk factors for CCM and outcomes among patients with and without TTNtv (Table 4 and Tables VII through IX in the online-only Data Supplement). At diagnosis of CCM, the mean LVEF of patients with (34.9±7.4) and without TTNtvs (36.8±9.5; *P*=not significant) were comparable; however, patients with TTNtvs had more heart failure hospitalizations and atrial fibrillation (*P*=0.003 for each) than those without TTNtvs. Recovery occurred in both groups, although the final mean LVEF was more depressed in patients with TTNtvs (39.6±14.2 versus 48.9±10.8; *P*=0.03).

**Table 4.**
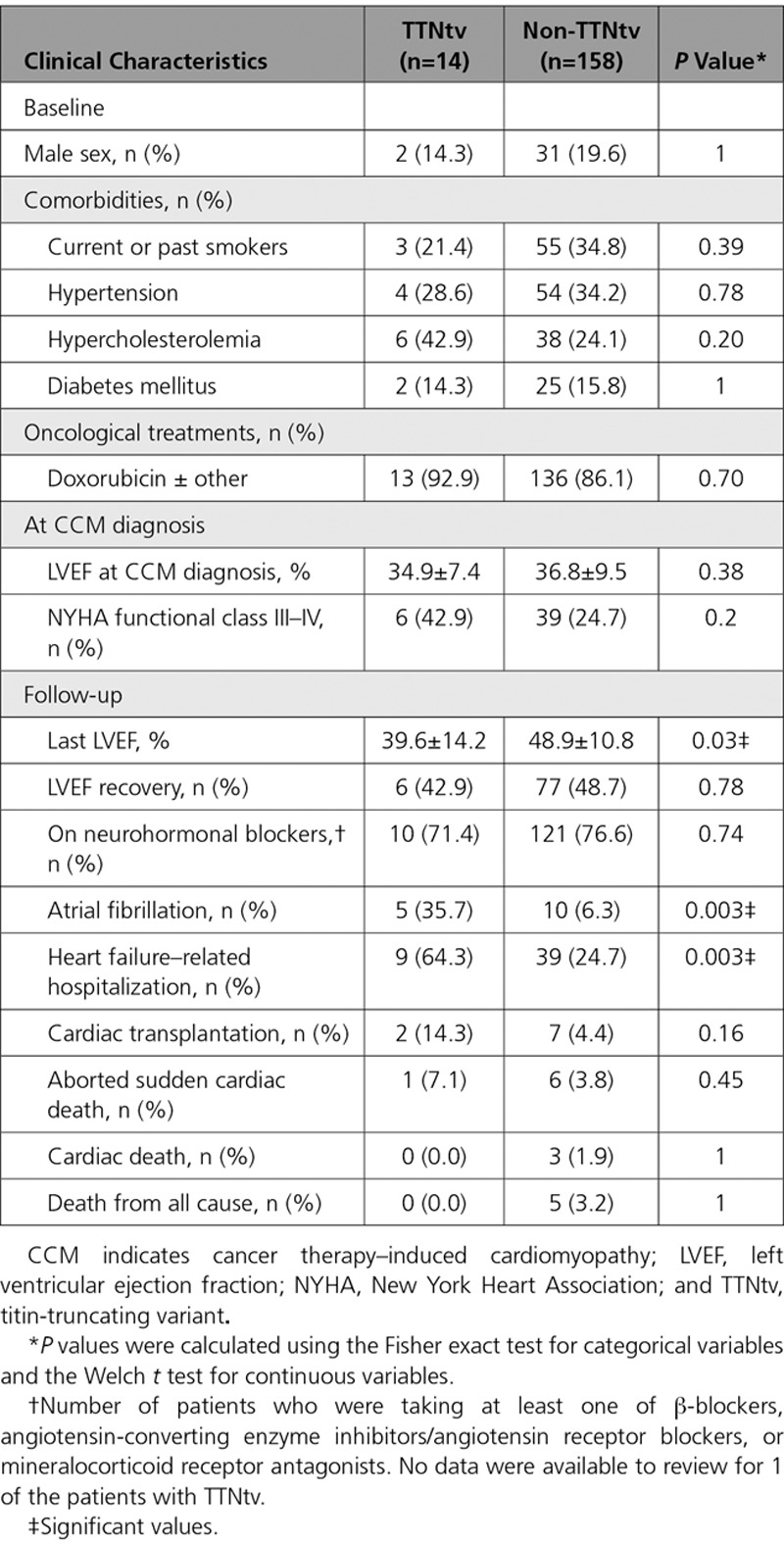
Comparisons of Risk Factors and Outcomes in Adult Patient Who Has CCM With and Without TTNtv

### Modeling CCM in TTNtv Mice

Given the multiple variables that can influence cardiotoxicity in human patients, we assessed whether TTNtvs increased susceptibility to anthracycline-induced cardiomyopathy in an experimental model. Doxorubicin was administered (3 doses of 5 mg/kg at weekly intervals; cumulative=45 mg/m^2^) to genetically identical mice, with the exception of the absence (wild-type) or presence (Ttn^tv/+^) of a heterozygous A-band titin truncation in one gene copy.^25,26^ Untreated Ttn^tv/+^ mice have normal LV function (not significantly different from wild-type mice) and anthracycline administration comparably depressed LV function in both genotypes at week 4 after treatments (Figure). However, at week 8, LV function recovered to baseline in wild-type mice but remained depressed through week 12 in Ttn^tv/+^ mice (*P*=0.0004 versus wild-type). Functional analyses in isolated cardiomyocytes confirmed that LV dysfunction reflected cell autonomous effects of anthracyclines (Figure B). Histological analysis of cardiac tissues from anthracycline-treated wild-type or Ttn^tv/+^ mice were comparable and showed no significant increase in fibrosis or apoptosis in comparison with untreated mice.

**Figure.**
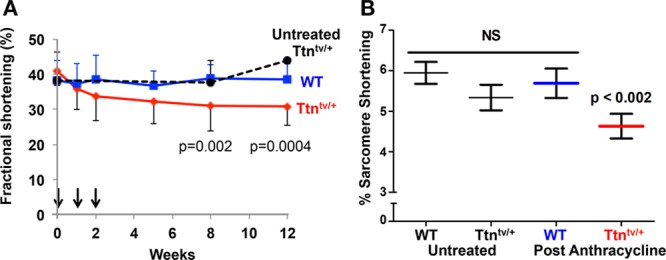
**Persistent cardiac dysfunction in Ttn^tv/+^ mice after anthracycline treatment. A**, Untreated Ttn^tv/+^ mice have left ventricular function comparable with wild-type (WT) mice.^25,26^ Intraperitoneal doxorubicin (5mg/kg) was administered (arrows) to WT and Ttn^tv/+^ mice (n=15 per genotype) in 3 successive weekly doses (cumulative dose=45 mg/m^2^). Serial echocardiograms showed persistent significantly depressed systolic function (mean fractional shortening ±SD) in Ttn^tv/+^ in comparison with WT mice (*P*=0.0004). **B**, Isolated cardiomyocytes (n≥52 per group) were studied 12 weeks after initial doxorubicin injection. Cardiomyocytes from doxorubicin-treated Ttn^tv/+^ mice had significantly depressed contractility (*P*<0.002) in comparison with cardiomyocytes from doxorubicin-treated WT mice or untreated mice. NS indicates not significant.

## Discussion

We demonstrate an increased prevalence of DCM-associated gene variants, predominantly TTNtvs, in adult patients who have cancer and pediatric patients who have acute myelogenous leukemia with CCM relative to controls. Although the majority of the patients with CCM have European ancestry, the frequency of cardiomyopathy variants in other patients with CCM who have other ancestries was not significantly different (Table II in the online-only Data Supplement). TTNtvs were identified in 16 of 213 CCM cases (7.5%), a considerably higher prevalence than in unselected breast and lung cancer TCGA participants (1.1%, *P*=7.36e–08) or healthy volunteers (0.7%, *P*=3.42e–06) and enriched in comparison with ancestry-matched reference populations (*P*=5.87e–14). Because cardiac status is not recorded for TCGA participants, these data provide conservative estimates of the burden of TTNtvs in CCM. Further support that TTNtvs contribute to CCM is derived from a mouse model of CCM: anthracycline-treated Ttn^tv/+^ mice and isolated cardiomyocytes had protracted LV and cellular dysfunction in comparison with wild-type.

Clinical outcomes among patients with CCM showed considerable variability, but cardiac function improved in 45% to 68% of adult and pediatric patients. Recovery occurred in 83 adults and 28 children, and was not significantly correlated (*P*≥0.5) with preexisting cardiovascular risk factors in adults, TTNtvs, high (>400 mg/m^2^) anthracycline dose, or trastuzumab therapy. However, adult patients who have CCM with TTNtvs had more heart failure hospitalizations and atrial fibrillation, as occurs in patients with DCM caused by TTNtvs,^33,34^ and cardiac function was worse in patients with than in patients without these variants.

In addition to TTNtvs, our analyses identified rare protein-altering variants in 5 genes previously studied in patients with DCM.^29^ Mutations in *BAG3*, *LMNA, MYH7*, and *TNNT2* are established autosomal dominant causes of DCM.^14,35^ *TCAP* mutations are occasionally identified in patients with DCM,^36^ but more commonly cause a recessive form of limb-girdle muscular dystrophy.^37^ Despite the low prevalence of variants in these genes across all CCM cohorts (4.7%), their critical roles in myocyte biology imply that variants identified here may contribute to an individual’s risk for CCM.

The increased burden of rare variants, including TTNtvs, indicate that genetics is an important component in CCM susceptibility and adverse outcomes. We demonstrate that genetics is associated with CCM susceptibility across different cancer types and treatment regimens, in particular, those including anthracycline and trastuzumab (Table 1). Genetic variants in previously identified cardiomyopathy genes were increased among adult cancer survivors with overt CCM and severe clinical courses, and among prospectively studied adult and pediatric patients with mild CCM identified during ongoing cancer treatment. It is notable that heart failure, cardiac transplantation, aborted sudden death, and cardiac death occurred years after completion of chemotherapy regimens in some patients with CCM (Table 4), an observation that underscores the need for continued cardiac surveillance in patients with CCM.

These data establish a genetic relationship between DCM and CCM. Cardiomyopathy variants were found in 12.2% of patients with CCM (Table 2), whereas these occur in ≈40% of patients with familial and sporadic DCM.^31,38–40^ Whether broader genomic analyses may uncover additional genetic contributors to CCM is worthy of study. TTNtvs are significantly prominent in DCM, occurring in 15% of ambulatory and 25% of end-stage patients,^30,31,34,38^ but are rarely identified in childhood-onset DCM,^41^ whereas here we identified TTNtvs in 8.1% of adults and 5.0% of children with CCM. TTNtvs found in patients with CCM, like those in patients with DCM, disrupted exons that are constitutively expressed in the heart and are overrepresented in the A-band (Table 3). TTNtvs also occur in ≈15% of patients with peripartum cardiomyopathy^21^ and in ≈10% of individuals with alcoholic cardiomyopathy,^42^ findings that imply additional cardiovascular stress can unmask the deleterious cardiac effects of TTNtvs. Consistent with this supposition, in vitro analyses of human isogenic cardiomyocytes (derived from induced pluripotent stems cells) demonstrate that titin provides an essential mechanical connection that propagates diastolic traction stresses from β-cardiac myosin during sarcomere formation. Cardiomyocytes with TTNtvs have diminished reassembly of sarcomeres after stress in comparison with cells without TTNtvs.^43^ We suggest that chemotherapy, like pregnancy and excessive alcohol, is an important provocation that is poorly tolerated by TTNtvs, a conclusion that is supported both by these human data and by analyses of anthracycline-treated TTNtv mice.

We recognize several limitations in this study. Given the demographic profiles of the cohorts studied here, further analyses of patients with diverse ancestries are needed. Cohort A was retrospectively recruited after diagnosis of CCM, and these patients had more severe phenotypes and longer durations of follow-up than the prospectively identified patients in cohorts B and C. Because breast cancer was the most common diagnosis in adult patients with CCM and all pediatric cases had acute myelogenous leukemia, these findings may not be relevant to other cancers and other treatment regimens. All patients had individual chemotherapy dosages and additional treatments based on clinical practice and treatment protocols. These and other variables may influence susceptibility to CCM. This study compared the frequency of TTNtvs among patients with CCM to the frequency of TTNtvs in a large cohort of patients with cancer participating in TCGA, some fraction of whom likely developed CCM. A limitation of our study is that we do not know which TCGA subjects developed CCM, potentially affecting the accuracy of the TTNtv frequency estimate in the CCM-free cancer cohort. A more ideal comparison group would have been patients treated with chemotherapy who did not develop cardiomyopathy. While recognizing these issues, we suggest that enrichment of protein-altering variants and TTNtvs across all cohorts strongly supports the conclusion that genetics, like high-dose anthracycline and combination therapy, and cardiovascular risk factors contribute to CCM.

Current strategies to diagnose CCM focus on imaging and circulating biomarkers^1,2,18,44–46^ and treatment guidelines are limited, often recommending interruption or discontinuation of chemotherapy that can negatively impact the survival of patients with cancer. The identification of genetic risk factors opens new opportunities to identify patients with cancer at high risk for CCM and to assess the efficacy of prophylactic cardioprotective drugs and treatment regimens.^47–49^ Future investigations will determine if early recognition of patients who have cancer with high CCM risk through genetic testing can optimize cancer and cardiovascular treatments to reduce CCM while providing effective cancer therapy.

## Acknowledgments

We thank the patients and physicians who participated in enrollment, in particular, Drs J. Segovia, C. Mitroi, M. Gomez-Bueno, F. Hernandez, A. Gamis, L. Sung, T. Alonzo, and S. Meshinchi. We also recognize many years of scientific guidance from Professor J. G. Puig.

## Sources of Funding

This work was supported in part by grants from the Instituto de Salud Carlos III (ISCIII; PI15/01551, PI17/01941, and CB16/11/00432 to Drs Garcia-Pavia and Alonso-Pulpon, and IFI17/00003 to Dr Restrepo-Cordoba), the Spanish Ministry of Economy and Competitiveness (SAF2015-71863-REDT to Dr Garcia-Pavia), the John S. LaDue Memorial Fellowship at Harvard Medical School (to Dr Kim), Wellcome Trust (107469/Z/15/Z to Dr Ware), Medical Research Council (intramural awards to Drs Cook and Ware; MR/M003191/1 to Dr Tayal), National Institute for Health Research Biomedical Research Unit at the Royal Brompton and Harefield National Health Service Foundation Trust and Imperial College London (to Drs Barton, Cook, and Ware), National Institute for Health Research Biomedical Research Centre at Imperial College London Healthcare National Health Service Trust and Imperial College London (to Drs O’Regan, Cook, Prasad, and Ware), Sir Henry Wellcome Postdoctoral Fellowship (to Dr Toepfer), Rosetrees and Stoneygate Imperial College Research Fellowship (to Dr Whiffin), Fondation Leducq (to Drs Cook, C.E. Seidman, and J.G. Seidman), Health Innovation Challenge Fund award from the Wellcome Trust and Department of Health (UK; HICF-R6-373; to Drs Cook, Barton, and Ware), the British Heart Foundation (NH/17/1/32725 to Dr O’Regan; SP/10/10/28431 to Dr Cook), Academy of Medical Sciences SGL015/1006 (to Dr de Marvao), Alex’s Lemonade Stand Foundation (to Dr Getz), National Institutes of Health (to Dr Aplenc: U01CA097452, R01CA133881, and U01CA097452; to Dr Arany: R01 HL126797; to Dr Ky: R01 HL118018 and K23-HL095661; to Dr J.G. Seidman and C.E. Seidman: 5R01HL080494, 5R01HL084553), and the Howard Hughes Medical Institute (to Dr C.E. Seidman). The Universitario Puerta de Hierro and Virgen de la Arrixaca Hospitals are members of the European Reference Network on Rare and Complex Diseases of the Heart (Guard-Heart; http://guard-heart.ern-net.eu). This publication includes independent research commissioned by the Health Innovation Challenge Fund (HICF), a parallel funding partnership between the Department of Health and Wellcome Trust. The Centro Nacional de Investigaciones Cardiovasculares (CNIC) is supported by the Ministry of Economy, Industry and Competitiveness and the Pro CNIC Foundation, and is a Severo Ochoa Center of Excellence (SEV-2015-0505). Grants from ISCIII and the Spanish Ministry of Economy and Competitiveness are supported by the Plan Estatal de I+D+I 2013–2016 – European Regional Development Fund (FEDER) “A way of making Europe.” The views expressed in this work are those of the authors, and the funding institutions played no role in the design, collection, analysis, or interpretation of the data or in the decision to submit the manuscript for publication.

## Disclosures

Drs C. E. and J. G. Seidman are founders and own shares in Myokardia Inc, a startup company that is developing therapeutics that target the sarcomere. James S. Ware receives grant support and honoraria from Myokardia. Myokardia had no involvement in this study. The other authors report no conflicts.

## Supplementary Material
