# Supplementary material for: Genetic Variants Associated With Cancer Therapy–Induced Cardiomyopathy
Source: Circulation. 2019 Apr 16;140(1):31–41. doi: 10.1161/CIRCULATIONAHA.118.037934 (PMC6613726; doi:10.1161/CIRCULATIONAHA.118.037934)
Supplement: Supplementary file 1 [file cir-140-31-s001.pdf]

Supplemental Material for online Publication to accompany:

Gene Variants Associated with Cancer Therapy-Induced Cardiomyopathy

Pablo Garcia-Pavia MD, PhD,*# Yuri Kim MD, PhD,* Maria Alejandra Restrepo-Cordoba MD,* Ida G. Lunde PhD, Hiroko Wakimoto MD, PhD, Amanda M. Smith, BA, Christopher N. Toepfer PhD, Kelly Getz PhD, Joshua Gorham MSc, Parth Patel MD, Kaoru Ito MD, PhD, Jonathan A. Willcox PhD, Zoltan Arany MD, PhD, Jian Li PhD, Anjali T. Owens MD, Risha Govind MSc, Beatriz Nuñez MD, Erica Mazaika MSc, Antoni Bayes-Genis MD, Roddy Walsh PhD, Brian Finkelman MD, PhD, Josep Lupon MD, PhD, Nichola Whiffin PhD, Isabel Serrano MD, William Midwinter BSc, Alicja Wilk BSc, Alfredo Bardaji MD, PhD, Nathan Ingold, Rachel Buchan MSc, Upasana Tayal PhD, MRCP, Domingo A. Pascual-Figal MD, PhD, Antonio de Marvao PhD, MRCP, Mian Ahmad PhD, Jose Manuel Garcia-Pinilla MD, PhD, Antonis Pantazis MD, Fernando Dominguez MD, PhD, A. John Baksi PhD MRCP, Declan P. O'Regan PhD, FRCR, Stuart D Rosen MD, FRCP, Sanjay K. Prasad MD, Enrique Lara-Pezzi PhD, Mariano Provencio MD, PhD, Alexander R. Lyon PhD, FRCP, Luis Alonso-Pulpon MD, PhD, Stuart A. Cook PhD, FRCP, Steven R. DePalma PhD, Paul J. R. Barton PhD,* Richard Aplenc MD, PhD,* Jonathan G. Seidman PhD,* Bonnie Ky MD, MSCE,* James S. Ware PhD, MRCP,* and Christine E. Seidman MD.*#

* These authors contributed equally

# Correspondence:

Pablo Garcia-Pavia, MD, PhD
Department of Cardiology
Hospital Universitario Puerta de Hierro
Manuel de Falla, 2; 28222 Madrid, Spain.
Phone: (+34) 91-191-7297
Fax: (+34) 91-191-7718
e-mail: pablogpavia@yahoo.es

Christine Seidman, MD
Departments of Medicine & Genetics
NRB Room 256
Harvard Medical School
77 Ave Louis Pasteur
Boston, MA 02115
Phone: 617-432-7871
Fax: 617-432-7830
Email: cseidman@genetics.med.harvard.edu

Supplemental Methods

CCM patients and healthy volunteers

Participants with cancer were selected for genetic analyses when CCM was identified, based on a decline in LVEF to <50% (cohort B) or <53% (cohorts A and C) and ≥10% reduction from baseline by echocardiography or <50% and ≥10% reduction from baseline by isotopic ventriculography. When pre-chemotherapy cardiac imaging was absent, patients were included when LVEF was ≤45% and no alternative cause for cardiac dysfunction other than chemotherapy was identified.

CCM patients in cohort A were retrospectively enrolled at cardiovascular clinics from six institutions in Europe: the Hospital Universitario Puerta de Hierro (Madrid, Spain), Hospital Universitario Virgen de la Arrixaca (Murcia, Spain), Hospital Universitari Germans Trias i Pujol (Barcelona, Spain), Hospital Clínico Universitario Virgen de la Victoria (Málaga, Spain), Hospital Universitario de Tarragona Joan XXIII (Tarragona, Spain), and the Royal Brompton and Harefield NHS Foundation Trust (London, UK). Physical evaluations, cardiac imaging, electrocardiograms, biomarkers and other studies that were obtained at the time of diagnosis of CCM or subsequently were performed as part of clinical care. Pre-chemotherapy studies were obtained from review of medical records. Eight patients with breast (n=5, treated with trastuzumab) or other solid tumor (n=3 treated with multiple agents) did not receive anthracyclines.

CCM patients in cohort B are prospectively enrolled breast cancer participants in the ongoing Penn Cardiotoxicity of Cancer Therapy (CCT) study (ClinicalTrials.gov NCT01173341) that was initiated in 2010 at the University of Pennsylvania Heath System. All participants are

over 18 years of age and had detailed phenotyping (echocardiography, electrocardiograms and clinical assessments) prior to and during and after cancer therapy initiation.[1] Treatment regimens were at the discretion of the oncology provider, and consisted of one of the following: 1) doxorubicin (240 mg/m^2) and cyclophosphamide, followed by paclitaxel; 2) doxorubicin (240 mg/m^2) and cyclophosphamide followed by paclitaxel and trastuzumab; or 3) cyclophosphamide or carboplatin with docetaxel and trastuzumab. Post-hoc quantitative assessment was performed on all echocardiograms in a blinded fashion (2-4 months) and then annually.

Cohort C patients are pediatric patients prospectively enrolled between June 2011 and April 2016 at multiple U.S. hospitals that participate in the Children's Oncology Group therapeutic trial for newly diagnosed acute myeloid leukemia (ClinicalTrials.gov NCT01371981). High-risk patients who had an appropriate allogeneic stem cell donor were scheduled to receive 342 mg/m^2 of anthracycline; non-high risk patients and high-risk patients without an appropriate allogeneic stem cell donor were scheduled to receive 492 mg/m^2 of anthracycline. All patients underwent detailed cardiac functional assessments that included either echocardiogram or multi-gated acquisition scans. Cardiac imaging was obtained prior to initiating each anthracycline course (Induction I, Induction II, Intensification II), prior to hematopoietic stem cell transplantation, at the end of protocol therapy, and every six to twelve months after protocol completion and at yearly intervals thereafter. LVEF and fractional shortening (FS) measurements were abstracted directly from the subset of submitted echo reports. Any discrepancy in toxicity grading between LVEF mode-specific measurements and the same echo report were reviewed and adjudicated by a cardiologist. In addition to the

routine adverse event monitoring, treating centers were required to submit LV fractional shortening and/or LVEF measurements at the end of each chemotherapy course and at the regular follow-up intervals described above.

The Cancer Genome Atlas[2] provides germline exome and genome data on a wide range of human cancers. From these data we extracted sequence data obtained on the Illumina platform (which was used to sequence CCM cohorts) from all participants with breast (TCGA-BRCA) and lung (TCGA-LUAD and TCGA-LUSD) cancers. TCGA participants with AML were not included as these were studied using a different sequencing platform. Sequence data was processed on the same pipeline (see below) used to analyze CCM cohorts. As anthracyclines therapy is effective and commonly prescribed for breast and lung cancer, we expect that most TCGA participants received this agent. The TCGA does not provide cardiac data. Based on the assumption that some TCGA participants developed CCM, comparison of the prevalence of rare cardiomyopathy gene variants between CCM cohorts studied here and TCGA participants provide a conservative estimate of the enrichment of cardiomyopathy variants in CCM.

Healthy volunteers of European ancestry were prospectively recruited by advertisement to the UK Digital Heart Project (https://digital-heart.org/) at the MRC-LMS, Imperial College London[3]. All participants reported no personal or family history of cardiovascular disease. All participants had clinical assessment and cardiac magnetic resonance imaging that confirmed normal cardiac structure and function.

Sequencing and genetic analyses:

Sequencing libraries were produced from genomic DNA extracted from CCM patients in cohort A and healthy volunteers and captured using the Illumina TruSight Cardio Sequencing kit.[3, 4] Sequencing libraries were produced from genomic DNA extracted from CCM patients in cohorts B and C using the Agilent XT2 kit and captured using a custom Agilent array (DCMv5) that contains all exons and flanking splice sequences for 71 putative DCM genes. All libraries were sequenced on Illumina platforms, reads were aligned to hg19 (GRCh37) using BWA-MEM and variants were identified using the Genome Analysis Tool kit (GATK) Haplotype-Caller tool[5]. Rare variants (defined as MAF<1.0e-4 in gnomAD[6]) were annotated using SnpEff (version4.3T).[7] For titin variants, analysis was further restricted to truncating variants in constitutively expressed exons (PSI>0.82 based on Genotype-Tissue Expression project (https://www.gtexportal.org) which corresponds to PSI>0.9 in our previous DCM analyses[3, 8]). Rare synonymous variants did not differ between any of the three cohorts or two sets of controls, confirming equivalent technical sensitivity of different platforms.

Two burden analyses were performed. In the first one, we compared the prevalence of rare variants in 9 DCM-causing genes (*TTN*, *DSP*, *MYH7*, *LMNA*, *TNNT2*, *TCAP*, *SNC5A*, *BAG3*, *TNNC1*) previously shown to be significantly associated with DCM in burden analyses of large-scale cohorts to the prevalence in the general population or to other cancer cohorts.[9] The frequencies of rare variants (MAF<1.0e-4) in ancestry-matched population data (gnomAD) were evaluated. Sub-analyses assessed variant frequencies in CCM patients and reference population stratified by ancestry. Rare variants were further characterized as loss-of-function or damaging missense, based on their predicted effects on protein function. The second analyses examined

all rare protein-altering variants the pre-specified nine genes plus 40 putative DCM genes contained in both TruSightCardio and the DCMv5 panel.

Assessment of cardiac function in mouse model system

Studies of wildtype (WT) and heterozygous littermate C57BL/6N mice with a titin A-band truncation ($Ttn^{tv/+}$)[10, 11] were conducted using protocols that were reviewed and approved by the Institutional Animal Care and Use Committee at Harvard Medical School (Boston, MA). WT and $Ttn^{tv/+}$ mice (n=15 per genotype) received weekly intra-peritoneal doxorubicin (5mg/kg) for three weeks (~45mg/m^2). Cardiac function of treated and untreated mice was assessed at baseline (age = 10-14 weeks) and then weekly using a digital ultrasound system (Vevo 2100 Imaging System and MS550D transducer; FujiFilm VisualSonics, Toronto, Canada) by an experienced observer blinded to mouse genotype and treatment. Mice were anesthetized under isoflurane vaporizer (VetEquip) and were placed on the heating table with ECG leads. Sedation was lightened after a mouse was positioned properly for the imaging, and all measurement were performed with heart rate between 500 and 550 beats per minute. Two-dimensional and M-mode images of the left ventricle (parasternal long axis and short axis) were obtained. Measurements were averaged from 3 consecutive heartbeats of M-mode tracings that include left ventricular end-diastolic (LVEDD) and end-systolic (LVESD) chamber dimensions, interventricular septal thickness (IVS) and left ventricular posterior wall thickness (LVPW). Left ventricular fractional shortening (FS) was calculated as (LVEDD - LVESD)/ LVEDD × 100 %.

Cardiomyocytes were isolated from untreated and doxorubicin-treated (12 weeks post first injection) wildtype and $Ttn^{tv/+}$ mice, as previously described.[12] Isolated cardiomyocytes were placed in wells of a 6-well plate that had been pre-coated with laminin. Laminin coating was performed for two hours before cardiomyocyte introduction at a concentration of 10 ug/mL in PBS (Composition: KH_2PO_4 1mM, NaCl 155 mM, Na_2HPO_4 3mM, at pH 7.4). Laminin coating solution was washed once with PBS before cells were introduced into the wells. Once cells were introduced they were left to incubate for 10 minutes to equilibrate to experimental temperature (30°C). Cells were imaged using a Keyence BZ-X710 microscope using a Nikon 40X/0.65 NA objective. Cells were kept at 30°C using microscope specific incubation chamber that was also used to deliver 20% O_2 and 5% CO_2 to the experimental chamber. Cells were paced at 1Hz using custom-built electrodes hooked up to a pacing unit (Pulsar 6i, FHC Brunswick, ME, USA) delivering 20V. Movies were acquired at 29 frames per second for 5 seconds (5 contractile cycles).

An ImageJ plugin SarCoptiM was used to track sarcomere lengths during contractile cycles.[13] Sarcomere tracking was then used to calculate cellular shortening (%), relaxed and contracted sarcomere lengths (µm), contractile cycle and relaxation durations (seconds).

Table S1. Burden analysis of 9 DCM genes in CCM cohorts compared to breast and lung cancer participants in The Cancer Genome Atlas (TCGA)

Gene	All CCM* (n=213)	Breast Cancer (n=1042)	Lung Cancer (n=1011)	p-value (all CCM vs. breast CA)	p-value (all CCM vs. lung CA)	p-value (all CCM vs. both CA)
BAG3	3 (1.4%)	8 (0.8%)	10 (1.0%)	0.41 (1)	0.48 (1)	0.44 (1)
DSP	0 (0.0%)	3 (0.3%)	1 (0.1%)	1 (1)	1 (1)	1 (1)
LMNA	1 (0.5%)	8 (0.8%)	8 (0.8%)	1 (1)	1 (1)	1 (1)
MYH7	3 (1.4%)	22 (2.1%)	13 (1.3%)	0.79 (1)	0.75 (1)	1 (1)
SCN5A	0 (0.0%)	1 (0.1%)	2 (0.2%)	1 (1)	1 (1)	1 (1)
TCAP	2 (0.9%)	0 (0.0%)	2 (0.2%)	0.03 (0.27)	0.14 (1)	0.05 (0.45)
TNNC1	0 (0.0%)	0 (0.0%)	1 (0.1%)	1 (1)	1 (1)	1 (1)
TNNT2	1 (0.5%)	4 (0.4%)	3 (0.3%)	1 (1)	0.54 (1)	0.55 (1)
TTN	16 (7.5%)	9 (0.9%)	13 (1.3%)	1.39e-07 (1.25e-06)	3.40e-06 (3.06e-05)	7.36e-08 (6.62e-07)
8 Genes (no TTN)	10 (4.7%)	46 (4.4%)	40 (4.0%)	0.86	0.57	0.72
9 Genes	26 (12.2%)	55 (5.3%)	53 (5.2%)	5.89e-04	5.68e-04	1.98e-04

*All CCM refers to all subjects from cohorts A, B, and C (Table 1).

The results shown here are in part based upon data generated by the TCGA Research Network.[2] All breast and lung cancer participants in the TCGA were included, irrespective of cancer subtypes, cancer treatments, or the presence or absence of CCM. As such, comparisons with CCM cohorts studied here reflect a conservative estimate of the enrichment of rare protein-altering variants in CCM. p-values were calculated via Fisher's exact test and their Bonferroni correction is noted in parenthesis.

Types of variants analyzed for each genes: all protein-altering variants (BAG3, LMNA, TCAP, TNNC1, TNNT2), missense variants and inframe deletion or insertion only (MYH7), frameshift variant, stop-gained, splice-donor and splice-acceptor variants only (DSP, SCN5A, TTN)

Table S2. Frequency of rare TTNtv and other pathogenic variants in CCM subjects stratified by non-Finnish European (NFE), African, and Asian ancestry

Ancestry	Non-Finnish European (NFE)			African			Asian		
Group	AC CCM (n=170)	AF gnomAD	pval	AC CCM (n=32)	AF gnomAD	pval	AC CCM (n=7)	AF gnomAD	pval
BAG3	2	3.79E-03	0.37	1	5.84E-03	0.31	0	6.31E-03	1
LMNA	0	3.12E-03	1	0	4.00E-03	1	0	3.48E-03	1
TCAP	1	7.49E-04	1	0	8.66E-04	1	0	2.72E-03	1
TNNC1	3	1.32E-04	0.75	0	2.46E-04	1	0	5.98E-04	1
TNNT2	0	1.23E-03	1	0	3.44E-03	1	0	2.56E-03	1
MYH7	2	7.77E-03	1	0	6.83E-03	1	0	9.51E-03	1
DSP	0	4.04E-04	1	0	2.46E-04	1	0	5.98E-04	1
SCN5A	1	1.58E-04	0.34	0	1.94E-04	1	0	5.58E-05	1
TTN	12	2.63E-03	**2.03E-10**	1	2.77E-03	0.16	3	2.07E-03	**3.18E-06**

AC: allele count, AF: allele frequency, gnomAD: genome aggregation database, pval: nominal p-value- for the comparison of the AF in CCM group to the AF in the ancestry matched gnomAD population.

Table S3. Number of rare protein-altering variants in 49 putative DCM genes and their burden testing in CCM cohorts

Gene	Cohort A (n=99)	Cohort B (n=73)	Cohort C (n=41)	All CCM (n=213)	HVOL (n=445)	p-value (all CCM vs. HVOL)	p-value (all CCM vs. gnomAD)
ABCC9	0	0	0	0	5	0.18	0.18
ACTC1	0	0	0	0	0	1	1
ALMS1	4	0	1	5	20	0.20	0.01
ANKRD1	0	0	1	1	0	0.32	0.61
BAG3	1	2	0	3	4	0.69	0.48
CAV3	1	0	0	1	1	0.54	0.32
CRYAB	0	0	1	1	2	1	0.37
CSRP3	0	0	0	0	0	1	1
DES	2	1	0	3	4	0.69	0.12
DMD	3	2	1	6	6	0.22	0.21
DNAJC19	0	0	0	0	0	1	1
DSG2	0	0	1	1	4	1	0.39

DSP	5	2	2	9	14	0.50	0.58
DTNA	0	1	0	1	3	1	0.73
EMD	0	0	0	0	0	1	1
EYA4	0	0	0	0	5	0.18	0.41
FKRP	2	1	2	5	1	0.02	0.01
FKTN	1	1	0	2	3	0.66	0.67
FXN	0	0	0	0	1	1	1
HADHA	0	1	0	1	2	1	0.53
HFE	0	0	0	0	3	0.56	0.63
ILK	0	0	0	0	2	1	0.63
LAMA2	3	5	3	11	19	0.69	0.51
LAMA4	2	0	3	5	5	0.31	1
LAMP2	0	0	0	0	1	1	0.63
LDB3	1	0	0	1	2	1	0.38
LMNA	0	1	0	1	2	1	1

MYBPC3	0	4	1	5	6	0.35	0.82
MYH6	2	5	0	7	9	0.42	0.69
MYH7	3	0	0	3	7	1	1
NEXN	2	1	0	3	5	0.72	0.44
PDLIM3	1	0	2	3	3	0.40	0.16
PLN	0	0	0	0	0	1	1
RBM20	3	1	1	5	7	0.54	0.62
SCN5A	1	4	1	6	7	0.37	0.83
SDHA	1	4	0	5	2	0.04	0.06
SGCB	0	1	0	1	2	1	0.63
SGCD	0	0	0	0	2	1	1
SGCG	3	1	0	4	1	0.04	0.02
TAZ	0	0	0	0	1	1	1
TBX20	0	0	0	0	1	1	1.00
TCAP	1	0	1	2	0	0.10	0.07

TMPO	0	0	0	0	2	1	0.08
TNNC1	0	0	0	0	0	1	1
TNNI3	0	0	0	0	2	1	1
TNNT2	0	1	0	1	0	0.32	0.50
TPM1	1	0	4	5	5	0.31	0.003
TTN*	44	16	16	76	120	0.04	0.85
VCL	1	0	2	3	7	1	0.49

*Only rare variants in TTN exons with PSI≥0.86 (excluding Novex-3) were considered in this analysis. There was no significant difference in the prevalence of all rare protein-altering variants (MAF<1.0e-4) or variants predicted as damaging in CCM patients in each cohort or the combined CCM cohort compared to healthy volunteers or in ancestry-matched reference populations.

Table S4. Summary of rare protein-altering variants in 49 putative DCM genes and their burden testing in CCM cohorts

	Cohort A	Cohort B	Cohort C	All CCM	HVOL	p-value	p-value
TTNtv* only	10	4	2	16	3	4.04e-06	1.58e-09
Any TTN* variants	44	16	16	76	120	0.04	0.85
All genes except TTN	44	39	27	110	178	0.02	0.39
All genes including TTNtv* only	54	43	29	126	181	0.0003	0.59
All genes including any TTN* variants	88	55	43	186	298	0.0004	0.31

*Only rare variants in TTN exons with PSI≥0.86 (excluding Novex-3) were considered in this analysis.

Table S5A. Loss-of-function (LoF) variants in putative 49 DCM genes in CCM cohorts

Gene	Variant*	Impact	Number of variants (n=213)	p-value (vs. gnomAD)	pLI
ALMS1	chr2:73613052 (c.57_58delGG)	Frameshift (p.Glu20fs)	1	1	0.00
DMD	chrX:32472779 (c.3602_3603insAAAA)	Frameshift (p.Arg1202fs)	1	0.16	1.00
FKTN	chr9:108382216 (c.1046T>C)	Splicing	1	0.21	0.00
LAMA4	chr6:112435865 (c.5206+1G>A)	Splicing	1	0.4	0.00
SDHA	chr5:225997 (c.457-1G>A)	Splicing	1	0.18	0.00
TBX20	chr7:35284677 (c.546-8T>A)	Splicing	1	0.005	0.34
TPM1	chr15:63336348 (c.363_364insTGAAGCT)	Frameshift (p.Asp122fs)	1	0.13	0.80
TTN	chr2:179399704 (c.101638G>T)	Nonsense (p.E33880X)	1		
TTN	chr2:179400742 (c.100731dupA)	Frameshift (p.Ser33578IlefsTer15)	1		
TTN	chr2:179410112 (c.95722+2delT)	Splicing	1		
TTN	chr2:179414849 (c.91715dupA)	Frameshift (p.Asn30572fs)	1	1.89e-09	0.00
TTN	chr2: 179422284 (c.60512-2A>C)	Splicing	1		
TTN	chr2:179425091 (c.85768C>T)	Nonsense (p.R28590X)	1		
TTN	chr2:179428124 (c.82734dupA)	Frameshift (p.Val27579SerfsTer15)	1		

TTN	chr2:179432234 (c.78625G>T)	Nonsense (p.E26209X)	1
TTN	chr2:179435679 (c.75179delA)	Frameshift (p.Asn25060fs)	1
TTN	chr2:179441250 (c.69715+6T>C)	Splicing	1
TTN	chr2:179446855 (c.66240delA)	Frameshift (p.Asp22081MetfsTer31)	1
TTN	chr2:179453355 (c.63096delT)	Frameshift (p.His21032fs)	1
TTN	chr2:179478777 (c.49345+2T>C)	Splicing	1
TTN	chr2:179571683 (c.29042-2A>C)	Splicing	1
TTN	chr2:179604819 (c.13141G>T)	Nonsense (p.E4381X)	1
TTN	chr2:179631116 (c.9693_9694delCT)	Frameshift (p.Tyr3232CysfsTer5)	1

Table S5B. Loss-of-function (LoF) variants in TTN*, DCM genes excluding TTN, and in all genes among CCM cohorts

	Cohort A (n=99)	Cohort B (n=73)	Cohort C (n=41)	All CCM (n=213)	p-value (all CCM vs. gnomAD)
TTN	10	4	2	16	1.89e-09
All LoF variants except TTN	2	2	3	7	0.61
All LoF variants	12	6	5	23	0.001

*Variants are defined based on the meta-transcript (LRG_391_t1 / ENST00000589042) that incorporates all exons in described TTN isoforms (including fetal and non-cardiac isoforms) with the exception exons that are unique to the novex transcripts. Only rare variants in TTN exons with PSI≥0.86 (and excluding Novex-3) were considered analyses.

Table S6. Characteristics of patients with CCM in the retrospective cohort (cohort A) based on presence or absence of identified rare protein-altering variants in 9 cardiomyopathy genes

Clinical characteristics of CCM patients in cohort A according to genetic variant status (n=99)					
	No variant (n=84)	TTNtv (n=10)	p-value*	Any rare variant (n=15)	p-value*
Baseline					
Age at chemotherapy onset – yr	49 ± 17.13	54.5 ± 8.05	0.09	46.73 ± 17.61	0.67
Male gender - n (%)	29 (34.5)	2 (20.0)	0.65	4 (26.6)	0.76
Family history of cardiomyopathy - n (%)	2 (2.4)	1 (10.0)	0.39	1 (6.6)	0.39
Family history of SCD - n (%)	3 (3.6)	1 (10.0)	0.48	1 (6.6)	0.48
Ethnicity - n (%)					
Caucasian	84 (100.0)	10 (100.0)	1	15 (100.0)	1
Comorbidities - n (%)					
Current or past smokers - n (%)	31 (36.9)	2 (20.0)	0.20	2 (13.3)	0.08
Hypertension - n (%)	26 (30.9)	3 (30.0)	0.62	3 (21.4)	0.54
Hypercholesterolemia - n (%)	21 (25)	5 (50.0)	0.18	6 (42.8)	0.20
Diabetes mellitus - n (%)	18 (21.4)	2 (20.0)	0.86	2 (14.2)	0.72

Oncological treatments - n (%)					
Anthracycline	76 (90.4)	10 (100.0)	0.73	15 (100)	0.60
Anti-HER2	19 (22.9)	1 (10.0)	0.56	1 (6.6)	0.29
TKIs	3 (3.6)	0 (0.0)	1	0 (0.0)	1
mTOR inhibitors	3 (3.6)	0 (0.0)	1	0 (0.0)	1
Cumulative anthracycline dose - mg/m2	271.4 ± 182.5	302.4 ± 75.6	0.43	290 ± 72.3	0.40
LVEF pre-chemotherapy - %	62.3 ± 5.6	61	0.85	60.6 ± 0.6	0.73
At CCM diagnosis					
Years from chemotherapy to CCM diagnosis	3 (1 - 9)	2.5 (0 - 8)	0.80	3 (0 - 8)	0.83
LVEF at CCM diagnosis - %	33.0 ± 10.3	32.8 ± 7.5	0.97	33 ± 8.1	0.84
NYHA functional class III–IV- n (%)	37 (44.0)	6 (66.0)	0.24	7 (50.0)	0.77
Sinus rhythm on ECG - n (%)	37 (44.0)	6 (66.0)	0.24	7 (50.0)	0.77
Atrial fibrillation on ECG - n (%)	73 (87.9)	7 (70.0)	0.30	12 (80.0)	0.52
Left bundle branch block on ECG - n (%)	7 (8.4)	2 (20.0)	0.30	2 (13.3)	0.52
Right bundle branch block on ECG - n (%)	13 (15.6)	0 (0.0)	0.68	1 (6.6)	0.67

Follow-up					
Duration of follow up – yr	4 (1 - 7)	4.5 (2 - 6)	0.72	5 (2 - 6)	0.44
NYHA functional class III–IV- n (%)	15 (18.0)	4 (40.0)	0.11	6 (40.0)	0.05
Lowest LVEF - %	31.1 ± 10.6	28.8 ± 7.9	0.67	28.3 ± 6.6	0.38
Last LVEF - %	49.0 ± 12.5	36.8 ± 14.2	0.02	38.1 ± 13.7	0.007
LVEF recovery[†] - n (%)	46 (54.7)	3 (30.0)	0.15	4 (26.6)	0.05
On neurohormonal blockers[‡] - n (%)	78 (92.9)	9 (100.0)	1	14 (100.0)	0.59
Atrial fibrillation - n (%)	8 (9.5)	5 (50.0)	0.004	7 (46.6)	0.001
Ventricular arrhythmias[§] - n (%)	6 (7.2)	2 (22.0)	0.14	3 (21.4)	0.11
Heart failure related hospitalization - n (%)	38 (45.2)	9 (90.0)	0.02	10 (67.0)	0.16
Cardiac transplantation - n (%)	6 (7.2)	2 (20.0)	0.16	3 (20.0)	0.13
Aborted sudden cardiac death - n (%)	4 (4.8)	1 (10.0)	0.02	3 (20.0)	0.06
ICD +/- CRT implant - n (%)	13 (15.4)	3 (30.0)	0.43	4 (26.6)	0.28
Cardiac death - n (%)	2 (2.4)	0 (0.0)	0.17	1 (6.6)	0.39
Death from all cause - n (%)	4 (4.7)	0 (0.0)	0.29	1 (6.6)	0.56

Data are expressed as numbers (%) or mean ± standard deviation except for time from chemotherapy to CCM diagnosis and duration of follow up, which are expressed as median (Q1 - Q3).

CCM, cancer therapy-induced cardiomyopathy; CRT, cardiac resynchronization therapy; ECG, electrocardiogram; HER2, human epidermal growth factor receptor 2; ICD, implantable cardioverter defibrillator; LVEF, left ventricular ejection fraction; mTOR, mammalian target of rapamycin; NYHA, New York Heart Association; TKIs, tyrosine kinase inhibitors

*p-values were calculated using Fisher´s exact test for categorical variables and Kruskal–Wallis test for continuous variables.

[†]LVEF recovery was defined as absolute increase in LVEF ≥5% with final LVEF ≥50% (or as a LVEF increase to the baseline value).

[‡]Number of patients, who were taking at least one of beta-blockers, angiotensin converting enzyme inhibitors/angiotensin receptor blockers, or mineralcorticoid receptor antagonists at the time of the latest follow-up. No data was available to review for one of patients with TTNtv.

[§]no data was available to review for one patient in TTNtv group and one patient in no variant group.

Table S7. Characteristics of patients with CCM in the prospective cohort (cohort B) based on presence or absence of identified rare protein-altering variants in 9 cardiomyopathy genes

Clinical characteristics of CCM patients in cohort B according to genetic variant status (n=73)					
	No variant (n=66)	TTNtv (n=4)	p-value*	Any rare variant (n=7)	p-value*
Baseline					
Age – yr	49.3 ± 11.0	51.0 ± 11.4	0.79	55.7 ± 7.2	0.26
Male gender - n (%)	0 (0.0)	0 (0.0)	1.00	0 (0.0)	1
Family history of cardiac disease- n (%)	41† (66.1)	2 (50.0)	0.61	4‡ (57.1)	1
Ethnicity - n (%)					
African American	22 (33.3)	1 (25.0)	1	1 (14.3)	0.42
Asian	3 (4.5)	1 (25.0)	0.21	1 (14.3)	0.34
Caucasian	41 (62.1)	2 (50.0)	0.64	5 (71.4)	1
Comorbidities - n (%)					
Current or past smokers	23 (34.8)	1 (25.0)	1	2 (28.6)	1
Hypertension	28 (42.4)	1 (25.0)	0.64	1 (14.3)	0.23
Hypercholesterolemia	16 (24.2)	1 (25.0)	1	1 (14.3)	1
Diabetes	7 (10.6)	0 (0.0)	1	0 (0.0)	1

Oncological treatments - n (%)					
Doxorubicin only	34 (51.5)	1 (25.0)	0.61	3 (42.9)	0.71
Anti-HER2 only	14 (21.2)	1 (25.0)	1	1 (14.3)	1
Doxorubicin plus anti-HER2	18 (27.3)	2 (50.0)	0.57	3 (42.9)	0.40
Cumulative anthracycline dose§ - mg/m2	240 ± 0	240 ± 0	n/a	240 ± 0	n/a
Radiation therapy	42 (63.6)	2 (50.0)	0.62	5 (71.4)	1.
LVEF pre-chemotherapy	55.2 ± 4.6	53.9 ± 4.9	0.64	54.0 ± 5.7	0.60
At CCM diagnosis					
Time from chemotherapy to CCM diagnosis - yr	0.7 (0.4 - 1.3)	0.6 (0.5 - 0.7)	0.01	1.0 (0.7 - 1.0)	0.22
LVEF at CCM diagnosis	41.7 ± 5.4	39.9 ± 4.2	0.47	39.9 ± 7.3	0.54
Sinus rhythm on ECG[‖] - n (%)	28 (100)	n/a	n/a	2 (100)	1
Atrial fibrillation on ECG[‖] - n (%)	0 (0)	n/a	n/a	0 (0)	1
Left bundle branch block on ECG[‖] - n (%)	0 (0)	n/a	n/a	0 (0)	1
Right bundle branch block on ECG[‖] - n (%)	0 (0)	n/a	n/a	0 (0)	1

NYHA functional class III-IV- n (%)	1 (1.5)	0 (0.0)	1	0 (0.0)	1
Follow-up					
Duration of follow-up – yr	1.8 (0.7 - 3.7)	1.6 (1.2 - 2.1)	0.62	1.8 (1.1 - 2.0)	0.39
Lowest LVEF - %	40.5 ± 5.3	38.8 ± 5.6	0.58	40.0 ± 11.4	0.94
Last LVEF - %	49.0 ± 8.1	54.2 ± 6.5	0.21	50.4 ± 12.6	0.87
LVEF recovery[#] - n (%)	28 (42.4)	3 (75.0)	0.32	5 (71.4)	0.23
On neurohormonal blockers[**] - n (%)	36 (54.5)	1 (25.0)	0.34	3 (42.9)	0.70
Atrial fibrillation - n (%)	0 (0.0)	0 (0.0)	1	0 (0.0)	1
Ventricular arrhythmias - n (%)	0 (0.0)	0 (0.0)	1	0 (0.0)	1
Heart failure related hospitalization - n (%)	0 (0.0)	0 (0.0)	1	0 (0.0)	1
Cardiac transplantation - n (%)	0 (0.0)	0 (0.0)	1	0 (0.0)	1
Aborted sudden cardiac death - n (%)	0 (0.0)	0 (0.0)	1	0 (0.0)	1
ICD ± CRT implant - n (%)	0 (0.0)	0 (0.0)	1	0 (0.0)	1
Cardiac death - n (%)	0 (0.0)	0 (0.0)	1	0 (0.0)	1
Death from all cause - n (%)	0 (0.0)	0 (0.0)	1	0 (0.0)	1

Data are expressed as numbers (%) or mean ± standard deviation except for time from chemotherapy to CCM diagnosis and duration of follow up, which are expressed as median (Q1 - Q3).

AVNRT, atrioventricular nodal re-entry tachycardia; CCM, cancer therapy-induced cardiomyopathy; CRT, cardiac resynchronization therapy; ECG, electrocardiogram; HER2, human epidermal growth factor receptor 2; ICD, implantable cardioverter defibrillator LVEF, left ventricular ejection fraction; n/a, non-available; NYHA, New York Heart Association

*p-values were calculated using Fisher´s exact test for categorical variables and Welch’s t-test for continuous variables.

[†]41 with family history of cardiac disease, 21 with no family history, and 4 with unknown family history

[‡]4 with family history of cardiac disease, 2 with no family history, and 1 with unknown family history

[§]58 patients in cohort B, who underwent anthracycline treatment received 240mg/m^2.

[||]28, 0, and 2 EKGs at cardiotoxicity diagnosis were available for review for no variant, TTNtv variant, and any rare variant groups, respectively.

[#]LVEF recovery was defined as absolute increase in LVEF ≥5% with final LVEF ≥50% (or as a LVEF increase to the baseline value.

**Number of patients, who were taking at least one of beta-blockers, angiotensin converting enzyme inhibitors/angiotensin receptor blockers, or mineralcorticoid receptor antagonists. No data was available to review for one of patients with TTNtv.

Table S8. Characteristics of patients with CCM in the prospective pediatric cohort (cohort C) based on presence or absence of identified rare protein-altering variants in 9 cardiomyopathy genes

Clinical characteristics of pediatric AML patients with CCM according to genetic variant status (n=41)					
	No variant (n=38)	TTNtv (n=2)	p-value*	Any rare variant (n=3)	p-value*
Baseline					
Age – yr	10.6 ± 5.6	16 ± 1.4	0.02	12.7 ± 5.9	0.61
Male gender - n (%)	15 (39.5)	2 (100.0)	0.17	2 (66.7)	0.56
Ethnicity - n (%)					
American Indian or Alaska Native	1 (2.6)	0 (0.0)	1	0 (0.0)	1
African American	9 (23.7)	0 (0.0)	1	0 (0.0)	1
Asian	1 (2.6)	2 (100.0)	0.004	2 (66.7)	0.01
Caucasian	24 (63.2)	0 (0.0)	0.15	1 (33.3)	0.55
Unknown	3 (7.9)	0 (0.0)	1	0 (0.0)	1
Oncological treatments - n (%)					
Anthracycline	38 (100)	2 (100)	1	3 (100)	1
Cumulative anthracycline dose - mg/m2[†]	488.1 ± 23.4	492	0.32	492	0.32

LVEF pre-chemotherapy - %	64.6 ± 5.9	63.4 ± 7.7	0.85	62.6 ± 5.6	0.59
At CCM diagnosis					
LVEF at CCM diagnosis - %	44.8 ± 6.7	43.0 ± 10.5	0.85	44.7 ± 8.0	0.98
Years from chemotherapy to CCM diagnosis	0.3 (0.2 - 0.4)	0.4 (0.3 - 0.4)	0.65	0.3 (0.3 - 0.4)	0.49
Follow-up					
Duration of follow-up - yr	2.2 (1.0 - 3.2)	2.0 (1.6 - 2.4)	0.86	2.8 (2.0 - 3.6)	0.60
Lowest LVEF - %	40.7 ± 8.9	43.0 ± 10.5	0.81	44.7 ± 8.0	0.49
Last LVEF - %	54.6 ± 13.4	55.4 ± 6.8	0.90	56.3 ± 5.0	0.67
LVEF recovery[‡] - n (%)	26 (68.4)	1 (50)	1	2 (66.7)	1
Death from heart failure - n (%)	2 (5.6)	0 (0.0)	1	0 (0.0)	1

Data are expressed as numbers (%) or mean ± standard deviation except for time from chemotherapy to CCM diagnosis and duration of follow up, which are expressed as median (Q1 - Q3).

CCM, cancer therapy-induced cardiomyopathy; LVEF, left ventricular ejection fraction

*p-values were calculated using Fisher´s exact test for categorical variables and Welch’s t-test for continuous variables.

†Planned cumulative anthracycline dose (492mg/m^2 for 40 patients and 342mg/m^2 for one patient) is noted, and actual dose may vary as 17 patients underwent dose modification of unknown quantity during treatment period.

‡LVEF recovery was defined as absolute increase in LVEF ≥5% with final LVEF ≥53% (or as a LVEF increase to the baseline value

Supplemental References

1. Narayan HK, Finkelman B, French B, Plappert T, Hyman D, Smith AM, Margulies KB and Ky B. Detailed Echocardiographic Phenotyping in Breast Cancer Patients: Associations With Ejection Fraction Decline, Recovery, and Heart Failure Symptoms Over 3 Years of Follow-Up. *Circulation*. 2017;135:1397-1412.

2. http://cancergenome.nih.gov/.

3. Schafer S, de Marvao A, Adami E, Fiedler LR, Ng B, Khin E, Rackham OJ, van Heesch S, Pua CJ, Kui M, Walsh R, Tayal U, Prasad SK, Dawes TJ, Ko NS, Sim D, Chan LL, Chin CW, Mazzarotto F, Barton PJ, Kreuchwig F, de Kleijn DP, Totman T, Biffi C, Tee N, Rueckert D, Schneider V, Faber A, Regitz-Zagrosek V, Seidman JG, Seidman CE, Linke WA, Kovalik JP, O'Regan D, Ware JS, Hubner N and Cook SA. Titin-truncating variants affect heart function in disease cohorts and the general population. *Nat Genet*. 2017;49:46-53.

4. Pua CJ, Bhalshankar J, Miao K, Walsh R, John S, Lim SQ, Chow K, Buchan R, Soh BY, Lio PM, Lim J, Schafer S, Lim JQ, Tan P, Whiffin N, Barton PJ, Ware JS and Cook SA. Development of a Comprehensive Sequencing Assay for Inherited Cardiac Condition Genes. *J Cardiovasc Transl Res*. 2016;9:3-11.

5. Van der Auwera GA, Carneiro MO, Hartl C, Poplin R, Del Angel G, Levy-Moonshine A, Jordan T, Shakir K, Roazen D, Thibault J, Banks E, Garimella KV, Altshuler D, Gabriel S and DePristo MA. From FastQ data to high confidence variant calls: the Genome Analysis Toolkit best practices pipeline. *Curr Protoc Bioinformatics*. 2013;43:11 10 1-33.

6. http://gnomad.broardinstitute.org.

7. Cingolani P, Platts A, Wang le L, Coon M, Nguyen T, Wang L, Land SJ, Lu X and Ruden DM. A program for annotating and predicting the effects of single nucleotide polymorphisms, SnpEff: SNPs in the genome of Drosophila melanogaster strain w1118; iso-2; iso-3. *Fly (Austin)*. 2012;6:80-92.

8. Roberts AM, Ware JS, Herman DS, Schafer S, Baksi J, Bick AG, Buchan RJ, Walsh R, John S, Wilkinson S, Mazzarotto F, Felkin LE, Gong S, MacArthur JA, Cunningham F, Flannick J, Gabriel SB, Altshuler DM, Macdonald PS, Heinig M, Keogh AM, Hayward CS, Banner NR, Pennell DJ, O'Regan DP, San TR, de Marvao A, Dawes TJ, Gulati A, Birks EJ, Yacoub MH, Radke M, Gotthardt M, Wilson JG, O'Donnell CJ, Prasad SK, Barton PJ, Fatkin D, Hubner N, Seidman JG, Seidman CE and Cook SA. Integrated allelic, transcriptional, and phenomic dissection of the cardiac effects of titin truncations in health and disease. *Sci Transl Med*. 2015;7:270ra6.

9. Walsh R, Thomson KL, Ware JS, Funke BH, Woodley J, McGuire KJ, Mazzarotto F, Blair E, Seller A, Taylor JC, Minikel EV, Exome Aggregation C, MacArthur DG, Farrall M, Cook SA and Watkins H. Reassessment of Mendelian gene pathogenicity using 7,855 cardiomyopathy cases and 60,706 reference samples. *Genet Med*. 2017;19:192-203.

10. Lunde IGW, Burke MA, Sououlis V, Linke WA, Gorham J, Conner D, Christensen G, Seidman JG and Seidman CE. Titin A-band truncation in mice causes stress-induced dilated cardiomyopathy. *Eur Heart J*. 2015;17:31.

11. Lunde IGW, Burke MA, Sououlis V, Linke WA, Gorham J, Conner D, Christensen G, Seidman JG and Seidman CE. Proximal titin A-band truncation causes dilated cardiomyopathy in response to increased afterload in mice. *Eur Heart J*. 2015;36:521.

12. Toepfer CN, Wakimoto H, Garfinkel AC, McDonough B, Liao D, Jiang J, Tai A, Gorham J, Lunde IG, Lun M, Lynch TL, Sadayappan S, Redwood CS, Watkins H, Seidman JG and Seidman CE. MYBPC3 mutations cause Hypertrophic Cardiomyopathy by Dysregulating Myosin: Implications for Therapy . *Science Translational Medicine*. 2018; 11:eaat1199.

13. Pasqualin C, Gannier F, Yu A, Malecot CO, Bredeloux P and Maupoil V. SarcOptiM for ImageJ: high-frequency online sarcomere length computing on stimulated cardiomyocytes. *Am J Physiol Cell Physiol*. 2016;311:C277-83.
